# Arthroscopy-assisted reduction for Jacob type II pediatric humeral lateral condyle fractures: a clinical efficacy study

**DOI:** 10.3389/fped.2025.1634178

**Published:** 2025-12-18

**Authors:** Chen Zhikai, Liu Yuqing, Li Yifeng, Wang Jian, Jiang Tao, Jiang Lin, Zhu Fuping

**Affiliations:** 1 Sports Medicine Department, Liuyang Orthopedics Hospital, Liuyang, Hunan, China; 2 Foot and Ankle Orthopedics Department, The First Affiliated Hospital of Hunan University of Chinese Medicine, Changsha, Hunan, China

**Keywords:** elbow arthroscopy, minimally invasive, Jacob type II, lateral condylar fracture of humerus, pediatric

## Abstract

**Background:**

Minimally invasive approaches are being increasingly employed in pediatric orthopedic trauma surgery. Clinical practice has seen applications of minimally invasive techniques for fractures such as intercondylar eminence avulsion fractures, femoral shaft fractures, and humeral shaft fractures. However, open reduction remains the primary surgical approach for pediatric humeral lateral condyle fractures. Open reduction disrupts peripheral blood circulation and increases the risk of epiphyseal injury. Damage to the epiphysis may affect a child's growth and development; therefore, surgical approach selection requires careful consideration. Our institution has accumulated substantial experience in applying arthroscopic techniques to trauma management. Through clinical exploration, we have developed an approach for treating pediatric humeral lateral condyle fractures involving arthroscopic reduction under endoscopic guidance combined with Kirschner wire fixation, aiming to provide new insights for clinical treatment.

**Objective:**

To compare the efficacy of arthroscopic reduction vs. open reduction for Jacob type II pediatric humeral lateral condyle fractures.

**Methods:**

A retrospective study was conducted on 60 pediatric patients with Jacob type II humeral lateral condyle fractures treated at Liuyang Orthopedics Hospital between January 2021 and June 2022. The cases were divided into an Arthroscopic Group and an Open Reduction Group based on surgical approach. The Arthroscopic Group underwent arthroscopy-assisted reduction with Kirschner wire fixation, while the control group (Open Reduction Group) received open reduction with Kirschner wire fixation. Operative time, intraoperative blood loss volume, and incision length were compared between the two groups; Pre- and postoperative Visual Analog Scale (VAS) pain scores, C-reactive protein (CRP) levels, and erythrocyte sedimentation rate (ESR) were compared; To assess whether arthroscopic reduction offers advantages over open reduction for pediatric humeral lateral condyle fractures, outcomes including the Mayo Elbow Performance Score (MEPS) and Baumann angle were evaluated at 3 months postoperatively.

**Results:**

Incisions healed by first intention in both groups. No statistically significant difference was found in operative time between groups [(30.17 ± 8.342) min vs. (29.07 ± 9.340) min, *P* = 0.632]. Incision length was significantly shorter in the Arthroscopic Group [(2.07 ± 0.254) cm vs. (4.63 ± 0.809) cm, *P* = 0.000]. Intraoperative bleeding was significantly less in the Arthroscopic Group [(7.59 ± 1.167) mL vs. (11.83 ± 2.706) mL, *P* = 0.012]. Both groups showed reduced VAS scores postoperatively, with significantly better scores in the Arthroscopic Group (*P* = 0.000). Postoperative CRP and ESR levels increased in both groups compared to preoperative values, but the Open Reduction Group demonstrated significantly greater increases (*P* < 0.05). At 3 months postoperatively, the Arthroscopic Group showed superior Mayo Elbow Performance Scores (*P* = 0.013), while no significant difference was observed in Baumann angle measurements.

**Conclusion:**

Arthroscopic reduction for pediatric humeral lateral condyle fractures offers smaller incisions, reduced bleeding, attenuated inflammatory response, and is more conducive to postoperative functional recovery.

## 1 Introduction

Lateral condylar fractures of the humerus represent a relatively common type of fracture around the elbow joint in children, accounting for 12%–17% of all elbow fractures in the pediatric population (1) Jacob (2) classified lateral condylar fractures of the humerus into three types based on the displacement distance. A study by Vigil et al. (3) on pediatric lateral condylar fractures of the humerus found that among children with type II fractures treated conservatively with plaster cast immobilization, 28.6% developed secondary displacement while 36.2% eventually required surgical intervention due to secondary displacement. Therefore, for Jacob type II fractures, surgical management is clinically preferred (4). The conventional approach involves open reduction and internal fixation (5). A study by Sojib (6) on pediatric humeral lateral condyle fractures revealed that the postoperative excellent-good rate was 75% in children treated with open reduction and internal fixation. However, open reduction may cause excessive stripping of the periosteum and tissues around the elbow joint (7), potentially leading to prolonged fracture healing, nonunion, or even malunion. This increases the risk of elbow varus or valgus deformity (8), impacting cosmetic appearance. Meanwhile, open reduction is a significant contributor to postoperative elbow joint stiffness (9). Therefore, employing superior surgical techniques for managing pediatric humeral lateral condyle fractures is critically urgent. With the widespread application of arthroscopic instruments in shoulder and knee joints, excellent clinical outcomes have been achieved (10, 11). Previous research indicates that compared with open reduction, arthroscopic fracture reduction significantly reduces postoperative inflammatory indices (12). Regarding surgical exposure, arthroscopic fracture reduction demonstrates less invasiveness than open reduction, with reduced blood loss and fewer postoperative wound complications (13). However, the exploration of elbow arthroscopy remains limited. Therefore, we investigated whether internal fixation devices could be implanted under arthroscopic guidance for Jacob type II pediatric humeral lateral condyle fractures. A retrospective study analyzed 60 pediatric cases with lateral condylar fractures of the humerus treated between January 2021 and June 2022. With a 2-year follow-up period, satisfactory outcomes were achieved with minimal complications, as detailed below.

## 2 Materials and methods

### 2.1 General information

All cases were sourced from Liuyang Orthopedics and Traumatology Hospital. Sixty pediatric patients with humeral lateral condyle fractures admitted between January 2021 and June 2022 were enrolled and randomly divided using a random number table into a treatment group (*n* = 30) receiving open reduction internal fixation (ORIF) and a control group (*n* = 30) receiving arthroscopically assisted open reduction internal fixation. Statistical analysis was performed on clinical data from 60 pediatric patients with Jacob type II humeral lateral condyle fracture. Among them, 42 were male and 18 were female, with an age range of 1.92–10 years. The mean age was (5.25 ± 1.90) years in the treatment group and (4.80 ± 1.85) years in the control group. All included pediatric lateral condylar fractures were classified using the Jacob clinical classification system (14). All surgical procedures were performed by the same senior surgeon. This study was approved by our hospital's ethics committee [Approval No.: Pre-review (2023070512)]. All patients provided signed surgical consent forms preoperatively.

### 2.2 Patient selection criteria

Inclusion criteria: (1) Age 1–14 years (15); (2) Pediatric humeral lateral condyle fractures classified as Jacob type II (14); (3) Radiographic diagnostic criteria: fracture fragment displacement between 2 and 4 mm; (4) Good patient compliance and ability to cooperate with anesthesia.

Exclusion criteria: (1) Open, old, or pathological fractures; (2) Jacob classification type I or III (14); (3) Radiography: fracture fragment displacement >4 mm; (4) Concurrent ipsilateral elbow joint fractures; (5) Cardiopulmonary or neurological comorbidities rendering patients unable to tolerate surgery; (6) Poor compliance affecting efficacy evaluation or incomplete data affecting evaluation.

### 2.3 Treatment methods

Open Reduction Group: Pediatric patients underwent brachial plexus block combined with intravenous anesthesia. In the supine position, a pediatric tourniquet was applied to the upper third of the arm with pressure set at 250 mmHg. With the shoulder elevated and elbow flexed at 90°, a 5 cm Kocher approach was utilized. Dissection proceeded through the interval between the brachioradialis muscle and lateral head of triceps. Fracture fragments were elevated using towel clips to remove hematoma, followed by saline irrigation. An assistant maintained exposure by pronating and adducting the forearm while retracting the olecranon medially with surgical retractors. Simultaneously, the medial humeral condyle was digitally pressured to achieve maximal elbow varus, fully exposing the trochlea. Under direct visualization, fracture reduction was performed using towel clips on proximal and distal fragments until articular surface congruity was achieved. Three 1.5 mm Kirschner wires were drilled from the posterior aspect of the lateral humeral condyle at 30° in the sagittal plane and 60° in the coronal plane to engage the opposite cortex (16). C-arm fluoroscopy confirmed satisfactory fracture reduction and fixation placement. The wires were trimmed with ends buried subcutaneously. After confirming unrestricted elbow range of motion, the wound was irrigated and closed in layers.

Arthroscopic Group: Pediatric patients underwent combined brachial plexus block and intravenous anesthesia. Positioned supine, a pediatric tourniquet was applied to the upper third of the arm with pressure set at 250 mmHg. A mechanical pump system maintained joint distension at 35 mmHg water pressure. The affected forearm was connected to a Maquet mechanical traction device, maintaining shoulder abduction at 90° and elbow flexion at 90°. Arthroscopic instruments measuring 2.7 mm in diameter were utilized. The direct lateral portal was established at the soft spot between the lateral condyle, olecranon tip, and radial head. A 10 mL syringe was used to inject fluid into the joint cavity via this portal approach to create the elbow joint access. Then, an anterolateral approach was established 2 cm proximal and 1 cm anterior to the lateral epicondyle (17), through which the power system was inserted. After debriding the synovium and hematoma under arthroscopic visualization, the forearm was supinated with the elbow flexed and wrist extended to expose the lateral condylar fracture line of the humerus. Under arthroscopic guidance, the lateral condylar fragment was reduced using mosquito forceps until the trochlear articular surface was restored to a smooth contour. Following satisfactory reduction, three 1.5 mm Kirschner wires were drilled into the contralateral side at 30° in the sagittal plane and 60° in the coronal plane from the posterolateral aspect of the lateral condyle (16). C-arm fluoroscopy confirmed acceptable fracture reduction and internal fixation position. The wire ends were then processed and buried subcutaneously. After flexion and extension exercises, the surgical site was irrigated and closed in layers.

### 2.4 Postoperative management

Both groups of children used forearm sling suspension postoperatively. Five-finger grasping exercises were initiated after anesthesia wears off. Passive elbow flexion and extension exercises were gradually started the next day, moving gently to prevent resistance to activity from the children. Within 2 weeks postoperatively, passive flexion and extension of the elbow joint reached 90°. Active flexion and extension were gradually initiated after 2 weeks, and both active and passive movements reached 90° by 4 weeks. Continued active exercises were performed from 4 to 8 weeks postoperatively until normal flexion and extension of the affected elbow were restored. During the first three months after discharge, patients should return to our hospital for monthly follow-up visits. Anteroposterior and lateral radiographs of the elbow joint should be obtained at each visit to assess fracture healing progress and evaluate elbow joint mobility. At month 3, if x-ray films show obscured fracture lines with continuous trabeculae crossing the fracture line, the internal fixation device should be removed on an outpatient basis. If x-ray films at month 3 still show clearly visible fracture lines, we recommend continued retention of the internal fixation device. Patients should continue monthly outpatient follow-up until radiographs demonstrate complete disappearance of fracture lines with continuous trabeculae crossing the fracture site before removing the internal fixation device.

### 2.5 Outcome measures

#### 2.5.1 Surgical outcome indicators

The operative time (min), incision length (cm), and intraoperative blood loss volume (mL) were recorded for both groups of children.

#### 2.5.2 Pain assessment indicators

Visual Analog Scale (VAS) pain scores were recorded preoperatively and postoperatively; Children marked corresponding points on a 10 cm graduated scale based on pain perception before and after treatment, where 0 represented no pain and 10 represented extreme pain (17).

#### 2.5.3 Blood biochemical indices

Fasting venous blood samples (5 mL) were collected at 6:00 AM on the morning after hospital admission and at 6:00 AM on the first postoperative day, following a fasting period of over 10 h. C-reactive protein (CRP) was measured using non-anticoagulated whole blood samples via immunoturbidimetry (Beckman automated biochemical analyzer with Yonghe Yangguang test strips). Erythrocyte sedimentation rate (ESR) was determined by Westergren method using anticoagulated whole blood (Sekexide automated ESR analyzer).

#### 2.5.4 Radiographic evaluation parameters

Recorded Banmann's angle at 3-month postoperative follow-up.

#### 2.5.5 Functional scoring metrics

Documented Mayo Elbow Performance Score (MEPS) and other functional indicators at 3-month postoperative follow-up (18). The Mayo Elbow Performance Score (MEPS) is primarily utilized to assess the severity of elbow joint dysfunction in patients, analyze treatment efficacy, and compare the merits of different therapeutic approaches. Excellent: ≥90 points; Good: 75–89 points; Fair: 60–74 points; Poor: <60 points.

### 2.6 Statistical analysis

Statistical analysis was performed using SPSS version 21.0 software. Measurement data are presented as mean ± standard deviation $\left(\bar{x}\pm s\right)$. For normally distributed data, intergroup comparisons were conducted using the independent samples *t*-test, while intragroup comparisons employed the paired samples *t*-test. Non-normally distributed data were analyzed using non-parametric tests. Intergroup comparisons of categorical data were performed using the *χ*² test, with a *P*-value < 0.05 considered statistically significant.

## 3 Results

All 60 pediatric patients were followed up for 1.08–2.17 years, with no instances of wound infection occurring in any case. At the 3-month follow-up, fracture lines had disappeared in all 60 patients, and internal fixation devices were removed during outpatient visits. Four patients in the Open Reduction Group developed elbow joint varus deformity. One patient exhibited limited extension (10°) with normal flexion range postoperatively. One patient presented with localized bony prominence on the lateral aspect of the elbow. Six patients reported dissatisfaction with scar appearance.

### 3.1 Baseline characteristics

No statistically significant differences were observed between the two groups regarding age, gender, body mass index (BMI), affected side, and time from injury to surgery (see Table 1).

**Table 1:** Comparison of baseline characteristics between groups.

Group	Cases	Age	Gender	BMI	Affected Side	Time from Injury to Surgery
		（$\bar{x}\pm s$ years)	(Male/Female)	$\left(\bar{x}\pm s\right)$	(Left/Right)	$\left(\bar{x}\pm s,d\right)$
Arthroscopic Group	30	4.80 ± 1.853	21/9	14.89 ± 5.134	13/17	3.15 ± 1.12
Open Reduction Group	30	5.25 ± 1.905	21/9	15.24 ± 6.106	14/16	3.45 ± 0.89
Statistical Value		*t* = 0.933	X^2^ = 0.077	*t* = −0.917	*t* = 0.830	*t* = −0.723
*P* Value		*P* = 0.355	*P* = 0.781	*P* = 0.083	*P* = 0.214	*P* = 0.327

### 3.2 Comparison of surgical evaluation metrics

**Figure 1:**
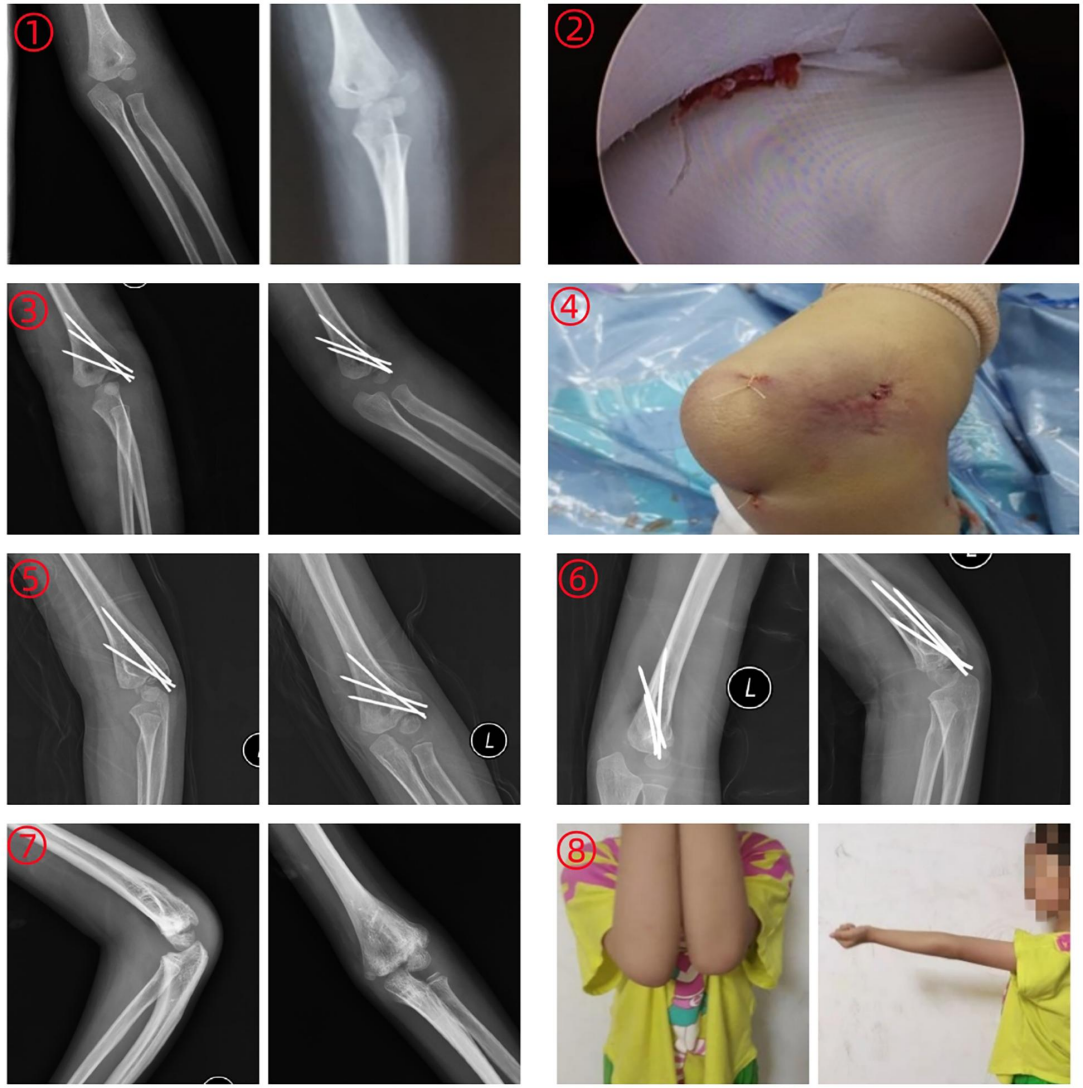
Female patient, 5 years old. (1): Preoperative anteroposterior and lateral radiographs of the left elbow joint showing lateral condylar fracture of humerus with posterolateral displacement of the distal fragment; (2): Intraoperative imaging following arthroscopic reduction of the lateral humeral condyle; (3): Postoperative review confirming anatomical reduction and proper fixation of the lateral humeral condyle fragment; (4): Postoperative incision appearance. (5): One-month postoperative x-ray review revealed satisfactory fracture reduction with callus formation at the fracture site. (6): Three-month postoperative x-ray review demonstrated continuous callus bridging the fracture line with obliteration of the fracture line, indicating fracture healing. (7): Two-year postoperative x-ray review confirmed bony union of the lateral condylar fracture of humerus without significant varus or valgus deformity. (8): Elbow joint functional assessment at 2-year follow-up.

There was no significant difference in operative time between the two groups (*t* = −0.481, *P* = 0.632). Regarding incision length, the arthroscopic surgery group had shorter incisions than the open reduction group, with a statistically significant difference (*t* = 16.586, *P* = 0.000). In terms of intraoperative blood loss volume, the arthroscopic group experienced less bleeding than the open reduction group, showing a statistically significant difference (*t* = 16.926, *P* = 0.012), as shown in Table 2 and Figure 1.

**Table 2:** Comparison of operative time, incision length, intraoperative bleeding, and postoperative Day 2 VAS scores between treatment and control groups $\left(\bar{x}\pm s\right)$.

Group	Cases	Operative Time (min)	Incision Length (cm)	Intraoperative Bleeding (mL)	Postoperative VAS Score
Arthroscopic Group	30	30.17 ± 8.342	2.07 ± 0.254	7.59 ± 1.167	2.30 ± 0.535
OpenReductionGroup	30	29.07 ± 9.340	4.63 ± 0.809	11.83 ± 2.706	3.23 ± 0.817
Statistical Group		*t* = −0.481	*t* = 16.586	*t* = 16.926	*t* = 5.234
*P* Value		*P* = 0.632	*P* = 0.000	*P* = 0.012	*P* = 0.000

### 3.3 Comparison of visual analog scale (VAS) scores

A statistically significant difference in VAS scores was observed between the two groups on postoperative day 2 (*t* = 5.234, *P* = 0.000), with arthroscopic surgery patients experiencing less pain (see Table 2).

### 3.4 Comparison of blood biochemical indices

The Arthroscopic surgery group exhibited significantly lower postoperative CRP and ESR levels [CRP: (*t* = 2.422, *P* = 0.019); ESR: (*t* = 4.822, *P* = 0.000)] with statistically significant differences (see Table 3).

**Table 3:** Comparison of postoperative CRP and ESR $\left(\bar{x}\pm s\right)$.

Group	Cases	Postoperative CRP (mg/L)	Postoperative ESR (mm/h)
Arthroscopic Group	30	7.464 ± 3.310	14.770 ± 4.462
Open Reduction Group	30	12.250 ± 10.305	22.770 ± 7.916
Statistical Group		*t* = 2.422	*t* = 4.822
*P* Value		*P* = 0.019	*P* = 0.000

### 3.5 Comparison of elbow joint function scores (Mayo) and radiographic parameters (Baumann angle)

No statistically significant difference was found in the 3-month Baumann angle measurements between the Arthroscopic surgery group and the Open Reduction group (*t* = 0.961, *P* = 0.217). The Arthroscopic surgery group demonstrated better functional outcomes in Mayo elbow scores (*t* = −2.576, *P* = 0.013), showing statistically significant differences (see Table 4).

**Table 4:** Comparison of elbow joint function scores and baumann angle at 3 months postoperatively $\left(\bar{x}\pm s\right)$.

Group	Cases	Mayo Score at 3 Months Postoperatively	Baumann Angle at 3 Months Postoperatively
Arthroscopic Group	30	96.155 ± 3.751	71.22 ± 6.31
OpenReductionGroup	30	92.077 ± 4.340	70.12 ± 4.65
Statistical Group		*t* = −2.576	*t* = 0.961
*P* Value		*P* = 0.013	*P* = 0.217

## 4 Discussion

The lateral condyle of humerus serves as the origin of the forearm extensor muscles (19). When a child falls with the forearm in supination and the elbow joint in adduction or abduction position, avulsion displacement by the forearm extensor tendons results in lateral condylar fracture of humerus (6). As the fracture line traverses the distal humeral epiphyseal plate and extends into the epiphysis, it is also classified as a Salter-Harris type IV epiphyseal fracture. Jacob classified pediatric humeral lateral condyle fractures into three types (20). Conservative treatment may be chosen for Jacob type I fractures since their fracture lines do not involve the articular surface (21). Open reduction is typically required for Jacob type III fractures to correct rotational displacement due to fragment rotation (22). Clinical controversy exists regarding whether open or closed reduction should be used for Jacob type II fractures with fragment displacement ranging from 2 mm to 4 mm (13). Some researchers (23) compared closed reduction percutaneous pinning with open reduction pinning for treating pediatric humeral lateral condyle fractures. They concluded there was no statistically significant difference in efficacy or safety between the two approaches. Moreover, closed reduction resulted in smaller scars and better postoperative cosmetic appearance. Another researcher reached similar conclusions using ultrasound-guided closed reduction for pediatric lateral condyle fractures (24). However, closed reduction requires multiple intraoperative fluoroscopies, prolonging operative time (24). Both ultrasound-assisted reduction and closed reduction are indirect reduction techniques, requiring fluoroscopy to evaluate the quality of bone fragment reduction. With the increasing adoption of arthroscopy and its successful application in the treatment of tibial intercondylar eminence avulsion fractures and humeral greater tuberosity avulsion fractures (25, 26), it offers a novel approach for managing pediatric humeral lateral condyle fractures.

This study revealed elevated postoperative inflammatory indices—including C-reactive protein (CRP) and erythrocyte sedimentation rate (ESR)—in both groups. However, the Arthroscopic Group demonstrated a less pronounced increase compared to the Open Reduction Group. Surgical intervention influences the hypothalamic-pituitary-adrenal axis hormone secretion, leading to localized inflammatory cell aggregation and immune response activation at the incision site. Consequently, this triggers elevation in postoperative inflammatory indices (27) Study (28) demonstrated that smaller surgical incisions result in lower postoperative inflammatory response, consistent with the findings of this research. The VAS pain score on postoperative day 2 was significantly lower in the Arthroscopic Group than in the Open Reduction Group. Study (29) documented intraoperative fluoroscopy frequency, hospitalization duration, and 3-month postoperative VAS pain scores to evaluate elbow arthroscopy-assisted reduction for pediatric humeral lateral condyle fractures. The Arthroscopic Group demonstrated significant advantages in fluoroscopy frequency and hospitalization duration compared to the open reduction approach. Both groups showed decreased VAS scores at 3 months postoperatively, with no significant difference observed between the arthroscopic and open reduction approaches. In this study, VAS scores were assessed on postoperative day 2, revealing superior pain scores (VAS) in the arthroscopic group compared to the open reduction group. This discrepancy is attributed to variations in the timing of pain assessment. Pain scores (VAS) demonstrated positive correlations with CRP and ESR levels, consistent with established relationships between pain perception and inflammatory mediators. An international study confirmed that nociceptive stimulation promotes inflammatory factor release from megakaryocytes and monocytes, leading to elevated inflammatory markers (30).

In this study, the intraoperative blood loss volume in the arthroscopic surgery group was 7.59 ± 1.167 mL, while that in the open reduction surgery group was 11.83 ± 2.706 mL. Arthroscopic surgery demonstrated significant advantages over the open reduction group in minimizing intraoperative blood loss. Hausman et al. (31) found that both open reduction and arthroscopy-assisted reduction can achieve anatomical reduction of fractures. However, arthroscopic reduction preserves local blood supply and minimizes surgical bleeding. Reduced irritation to soft tissues exerts a positive impact on preventing postoperative joint deformities (32). Arthroscopic surgery enables maximal visualization of intra-articular structures within a confined space, avoiding extensive tissue dissection and thereby significantly reducing intraoperative blood loss. A domestic retrospective study comparing open surgery with arthroscopic surgery indicated: arthroscopic procedures effectively reduce intraoperative bleeding (33), consistent with our findings.

At the 3-month postoperative follow-up, the Arthroscopic Group demonstrated superior Mayo Elbow Performance Scores compared to the Open Reduction Group. Arthroscopic surgery reduces tissue irritation and lowers postoperative pain scores (32). This pain reduction facilitates earlier initiation of postoperative rehabilitation (34). Kyle et al. (35) similarly found that elbow joint function improvement after elbow arthroscopy was superior to the open reduction group, with a lower complication rate. This finding is consistent with the present study.

Additionally, it is noteworthy that international researchers have attempted arthroscopy-assisted reduction for pediatric humeral lateral condyle fractures. After closed reduction, arthroscopic examination revealed inadequate reduction in a quarter of pediatric patients, necessitating conversion to open reduction (36). Therefore, using arthroscopic assessment solely as an evaluation tool after closed reduction increases procedural uncertainty. To avoid such situations, we performed reduction under arthroscopic guidance during surgery, adjusted the reduction under arthroscopic visualization, and eliminated repeated attempts that could cause cartilage damage. The limited space within the elbow joint cavity and the complexity of the procedure require surgeons to possess proficient arthroscopic skills, detailed knowledge of local anatomy, and extensive experience in open reduction (37), resulting in a steep learning curve. The limitation of this study lies in its small sample size. Future research should increase the sample size to enhance the stability of the findings. As children age, epiphyses develop, and the closure of the physeal line necessitates further prospective randomized studies and long-term follow-up to validate whether elbow joints develop varus or valgus deformities.

In summary: Arthroscopy-assisted reduction offers smaller incisions, reduced postoperative pain, less intraoperative bleeding, lower postoperative inflammatory indices, and faster early postoperative recovery, merits widespread clinical adoption.

## Data Availability

The original contributions presented in the study are included in the article/Supplementary Material, further inquiries can be directed to the corresponding author.
